# Safety and tolerability of artemether-lumefantrine versus dihydroartemisinin-piperaquine for malaria in young HIV-infected and uninfected children

**DOI:** 10.1186/1475-2875-8-272

**Published:** 2009-11-30

**Authors:** Shereen Katrak, Anne Gasasira, Emmanuel Arinaitwe, Abel Kakuru, Humphrey Wanzira, Victor Bigira, Taylor G Sandison, Jaco Homsy, Jordan W Tappero, Moses R Kamya, Grant Dorsey

**Affiliations:** 1 Oregon Health and Science University, Portland, USA; 2 Department of Epidemiology, University of California, Berkeley, USA; 3 Makerere University - University of California San Francisco Research Collaboration, Kampala, Uganda; 4 Department of Medicine, University of Washington, Seattle, USA; 5 Institute for Global Health, University of California, San Francisco, USA; 6 Global AIDS Program, Centers for Disease Control and Prevention, Atlanta, USA; 7 Department of Medicine, Makerere University Medical School, Kampala, Uganda; 8 Department of Medicine, University of California, San Francisco, Box 0811, San Francisco, CA 94143, USA

## Abstract

**Background:**

Artemisinin combination therapy has become the standard of care for uncomplicated malaria in most of Africa. However, there is limited data on the safety and tolerability of these drugs, especially in young children and patients co-infected with HIV.

**Methods:**

A longitudinal, randomized controlled trial was conducted in a cohort of HIV-infected and uninfected children aged 4-22 months in Tororo, Uganda. Participants were randomized to treatment with artemether-lumefantrine (AL) or dihydroartemisinin-piperaquine (DP) upon diagnosis of their first episode of uncomplicated malaria and received the same regimen for all subsequent episodes. Participants were actively monitored for adverse events for 28 days and then passively for up to 63 days after treatment. This study was registered in ClinicalTrials.gov (registration # NCT00527800).

**Results:**

A total of 122 children were randomized to AL and 124 to DP, resulting in 412 and 425 treatments, respectively. Most adverse events were rare, with only cough, diarrhoea, vomiting, and anaemia occurring in more than 1% of treatments. There were no differences in the risk of these events between treatment groups. Younger age was associated with an increased risk of diarrhoea in both the AL and DP treatment arms. Retreatment for malaria within 17-28 days was associated with an increased risk of vomiting in the DP treatment arm (HR = 6.47, 95% CI 2.31-18.1, p < 0.001). There was no increase in the risk of diarrhoea or vomiting for children who were HIV-infected or on concomitant therapy with antiretrovirals or trimethoprim-sulphamethoxazole prophylaxis.

**Conclusion:**

Both AL and DP were safe and well tolerated for the treatment of uncomplicated malaria in young HIV-infected and uninfected children.

**Trial Registration:**

ClinicalTrials.gov: NCT00527800; http://clinicaltrials.gov/ct2/show/NCT00527800

## Background

Guidelines for the treatment of uncomplicated malaria in Africa have recently undergone a paradigm shift away from inexpensive monotherapies, such as chloroquine and sulphadoxine-pyrimethamine, to artemisinin-based combination therapies (ACT). Two of the most important artemisinin-based combinations for use in Africa are artemether-lumefantrine (AL) and dihydroartemisinin-piperaquine (DP). AL has been shown to be highly efficacious and well tolerated, and has become the most widely recommended first-line regimen in Africa [1,2]. DP, a newer ACT, has shown excellent efficacy in multiple trials from Asia and Africa and is considered a highly promising drug for global deployment due to its simple once-a-day dosing and extended post-treatment prophylactic effect [3-7]. Although ACT has been shown to be highly efficacious, concerns remain about its safety and tolerability, especially in certain patient populations, such as young children and those with co-morbidities such as HIV.

There are a number of methodological challenges in evaluating the safety of anti-malarial drugs, such as establishing causality between drugs and adverse events in the setting of malaria. The World Health Organization (WHO) has established guidelines for evaluating the efficacy of anti-malarial drugs, but no similar guidelines exist for safety monitoring [8-10]. A lack of uniformly applied methods for adverse events reporting limits the ability to compare data from different studies. Additionally, most anti-malarial safety data come from clinical trials evaluating single episodes of malaria, while in practice African children are often treated repeatedly for recurring episodes of malaria over a relatively short period of time. Despite these challenges, comparative data on the safety and tolerability of different forms of ACT are critical for making informed policy decisions.

Results of a study comparing the efficacy AL and DP for the treatment of uncomplicated malaria using a longitudinal study design in a cohort of young children living in an area of very high malaria transmission intensity in Uganda were recently published [11]. This cohort included HIV-unexposed (HIV-uninfected children born to HIV-uninfected mothers), HIV-exposed (HIV-uninfected children born to HIV-infected mothers), and HIV-infected infants enrolled from six weeks to 12 months of age and followed up to one year. Results presented here include detailed data on the safety and tolerability of these therapies with follow-up extended for two additional months, in the context of repeated treatments, HIV co-infection, and the concomitant use of trimethoprim-sulphamethoxazole (TS) prophylaxis and anti-retroviral (ARV) therapy.

## Methods

### Study site and subjects

This study was conducted in Tororo, a rural district in southeast Uganda, where malaria is holoendemic [12]. Participants were drawn from a cohort of HIV-unexposed, HIV-exposed, and HIV-infected children, enrolled between August 2007 and April 2008 using convenience sampling. Eligibility criteria included: (1) age six weeks to twelve months at enrollment; (2) documented HIV status of mother and child; (3) agreement to receive all medical care at the study clinic; (4) agreement to avoid medication administered outside the study; and (5) no history of allergies to AL or DP. All study participants were given a long-lasting insecticide-treated bed net (ITN) at enrollment. Daily TS prophylaxis was given to all HIV-infected and HIV-exposed participants, as per national guidelines. ARV therapy (nevirapine + lamivudine + zidovudine or stavudine) was provided to all HIV-infected participants meeting standardized WHO criteria. The study protocol was approved by the Uganda National Council of Science and Technology and the institutional review boards of the University of California, San Francisco, Makerere University, the University of Washington, and the Centers for Disease Control and Prevention.

### Follow-up of study participants, malaria diagnosis and treatment

Details of the study procedures have been published previously and are summarized here [11]. Study participants were followed for all of their health needs in a dedicated study clinic. Children presenting with new medical problems underwent standardized evaluation. Subjects who presented with fever had blood obtained by fingerprick for a thick blood smear. If the thick blood smear was positive, the patient was diagnosed with malaria. Study participants ≥4 months of age and weighing ≥5 kg were randomly assigned to receive either open label AL or DP at the time their first episode of uncomplicated malaria was diagnosed. Study participants received the same treatment regimen for all subsequent episodes of uncomplicated malaria diagnosed during the study.

Study drugs were administered according to weight-based guidelines for fractions of tablets as follows: AL (Coartem^®^, Novartis, 20 mg artemether/120 mg lumefantrine tablets), administered as one (5-14 kg) or two (15-24 kg) tablets given twice daily for three days; DP (Duocotecxin^®^, Holley Pharm, 40 mg dihydroartemisinin/320 mg piperaquine tablets), targeting a total dose of 6.4 and 51.2 mg/kg of dihydroartemisinin and piperaquine, respectively, given in three equally divided daily doses to the nearest 1/4 tablet.

### Definitions of adverse events

Participants randomized to treatment with study medications were assessed for adverse events beginning after their first dose of study drug. An adverse event was defined as any untoward medical occurrence, irrespective of its suspected relationship to the study medications as per International Conference of Harmonization (ICH) guidelines [13] and graded according to severity (mild, moderate, severe, life threatening). A pre-determined list of 43 adverse events and standardized definitions was developed using the United States National Institutes of Health, Division of AIDS Table for Grading the Severity of Adult and Pediatric Adverse Events [14]. A serious adverse event was defined as any adverse experience that resulted in death, life threatening experience, participant hospitalization, persistent or significant disability or incapacity, or specific medical or surgical intervention to prevent serious outcome.

### Active and passive surveillance for adverse events

On the day that treatment was initiated (day 0), a baseline assessment was conducted, consisting of a standardized history and physical examination and haemoglobin measurement using a portable spectrophotometer (Hemocue, Angelholm Sweden). Active surveillance for adverse events following therapy consisted of assessment on days 1, 2, 3, 7, 14, 21, 28 and any other day they felt ill. Haemoglobin measurements were repeated on day 28 or earlier if severe anaemia was suspected. At each follow-up visit, adverse events were identified by evaluating for any new or worsening symptoms, physical exam findings, or haemoglobin values, as compared to the day 0 baseline assessment. Participants with abnormalities present on day 0 were not classified as experiencing an adverse event unless the symptom worsened from baseline, or resolved and then recurred. Following the initial 28-day active surveillance period, participants were asked to return to the clinic only when they desired medical attention. Passive surveillance for adverse events continued until the participant was re-treated with study drugs (at which time a new cycle of adverse event assessment began), the end of the study period (September 2008), or withdrawal from the study.

### Statistical analysis

Data were double entered into Microsoft Access and analysed using Stata version 10 (Stata, College Station, TX, USA). Data were evaluated with an intention-to-treat analysis including all episodes of uncomplicated malaria in children who were assigned treatment with study drugs through September 2008. The cumulative risks of the first occurrence of individual adverse events following the initiation of study drug therapy were estimated using the Kaplan-Meier product limit formula. Participants were censored on the day prior to repeat therapy with study drugs to remove the potential confounding effect of recurrent malaria, which can mimic adverse events [15]. Cumulative risks were estimated over the following pre-specified time intervals following the initiation of therapy; days 1-3 and 4-28 during active surveillance and days 29-63 during passive surveillance considering only patients who were at risk during these time intervals. Analysis of adverse events during passive surveillance was limited to 63 days because this is typically the longest duration of follow-up used in anti-malarial clinical trials and likely captures the majority of adverse events causally related to study drugs [11]. The risks of individual adverse events between treatment arms were compared using Cox proportional hazard models, adjusting for repeated treatments for malaria in the same patient.

Multivariate Cox proportional hazards models were used to measure the associations between the following covariates and the risk of common adverse events after 28 days of follow-up, adjusting for repeated measures in the same patient: 1) age, 2) duration since last treatment with study drugs, and 3) combination of HIV status, concomitant TS use and ARV use. Comparisons of adverse events due to anaemia after 28 days of follow-up were modeled with a binomial distribution using generalized estimating equations, adjusting for repeated measures in the same study participant with exchangeable correlation and robust standard errors. A p-value < 0.05 was considered statistically significant.

## Results

### Patient population and characteristics of study drug treatments

**Figure 1:**
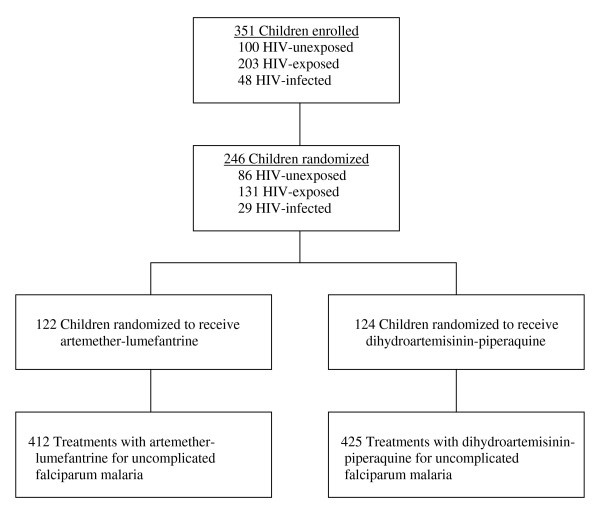
**Trial profile**. Enrollment and randomization of study patients.

A total of 351 children were enrolled in the cohort study, of which 246 (70%) had at least one episode of uncomplicated malaria and were randomized to study drugs. A total of 122 children were randomized to AL resulting in 412 treatments and 124 children were randomized to DP resulting in 425 treatments (Figure 1). Overall, 54% of the study drug treatments were given in children one year of age or younger, 38% of patients were taking TS concomitantly, 10% of patients were HIV infected, and 8% of patients were taking ARVs concomitantly (Table 1). Considering 28-day outcomes, 715 (85%) completed follow-up without recurrent malaria, 106 (13%) had recurrent malaria, and 16 (2%) had incomplete follow-up. Patients treated with AL were more likely to have been treated with study drugs in the previous 28 days compared to those treated with DP (18% vs. 4%) (Table 1). When follow-up was extended to 63 days, 492 (59%) patients had recurrent malaria, with no difference observed between those treated with AL and those treated with DP.

**Table 1:** Baseline characteristics of all episodes of uncomplicated malaria treated with study drugs

Characteristic	Treatment Group	
	
	AL (n = 412)	DP (n = 425)
Treatment episodes per child, median (range)	2 (1-11)	2 (1-9)
Age at time of treatment in months, mean (SD)	12.5 (3.6)	12.6 (3.8)
4-10 months, n (%)	104 (25%)	118 (28%)
>10-12 months, n (%)	111 (27%)	114 (27%)
>12-15 months, n (%)	85 (21%)	78 (18%)
>15-22 months, n (%)	112 (27%)	115 (27%)
HIV status, TS use, ARV use		
HIV-uninfected not taking TS, n (%)	248 (60%)	271 (64%)
HIV-uninfected taking TS, n (%)	115 (28%)	121 (28%)
HIV-infected only taking TS, n (%)	8 (1.9%)	8 (1.9%)
HIV-infected taking TS and ARVs, n (%)	41 (10%)	25 (5.9%)
Duration since last study drug treatment		
17 - 28 days, n (%)	76 (18%)	18 (4.2%)
28 - 63 days, n (%)	146 (35%)	192 (45%)
>63 days or no prior treatment, n (%)	190 (46%)	215 (51%)

### Comparison of risks for clinical adverse events between study drugs

Among the list of 42 clinical adverse events that were included in our standardized assessment, 32 were not reported, 7 (dyspnea, dysphagia, jaundice, malaise, rash, seizure, and weight-loss) occurred in less than 1% of study drug treatments, and 3 (cough, diarrhoea, and vomiting) were frequent enough for comparative analyses. Considering 63 days of follow-up among all 837 treatments with study drugs; 415 adverse events due to cough (373 mild and 42 moderate severity), 179 adverse events due to diarrhoea (168 mild, 10 moderate, and one severe), and 56 adverse events due to vomiting (all mild) were reported. Any adverse event due to cough, diarrhoea, or vomiting occurred in 296 of 412 (72%) treatments with AL and 313 of 425 (74%) treatments with DP. Comparisons of the risks for the three most common adverse events between the AL and DP treatment arms after 1-3, 4-28, and 29-63 days of follow-up are presented in Table 2. There were no statistically significant differences in the risks of these adverse events between the treatment arms for any time interval.

**Table 2:** Risk of common adverse events for discrete time periods following initiation of therapy

Time interval	Treatment arm	Adverse event								
		
		Cough			Diarrhoea			Vomiting		
		
		Risk*	HR^† ^(95% CI)	p-value	Risk*	HR^† ^(95% CI)	p-value	Risk*	HR^† ^(95% CI)	p-value
1-3 days	AL	7.8%	1.14 (0.71-1.84)	0.59	4.1%	1.13 (0.59-2.19)	0.71	1.2%	1.36 (0.41-4.44)	0.61
	DP	8.9%			4.7%			1.7%		

4-28 days	AL	39.8%	1.17 (0.93-1.48)	0.18	18.5%	0.81 (0.57-1.15)	0.23	2.1%	2.10 (0.92-4.79)	0.08
	DP	47.1%			15.1%			4.4%		

28-63 days	AL	51.9%	0.99 (0.68-1.46)	0.97	22.1%	0.90 (0.53-1.53)	0.70	11.6%	0.70 (0.34-1.43)	0.33
	DP	47.1%			18.9%			7.4%		

* Cumulative risk during time interval of interest estimated using the Kaplan-Meier product limit formula

^† ^HR = hazard ratio for DP vs. AL, generated using multivariate Cox proportional hazard models adjusted for repeated measures in the same patient

### Other risk factors for clinical adverse events

Non-treatment related risk factors for diarrhoea and vomiting over 28 days of follow-up stratified by treatment group were identified using multivariate analysis (Table 3). Risk factors of interest included age, duration since last treatment with study drugs, HIV status, concomitant TS use, and concomitant ARV use. Adverse events due to cough were not included in this analysis because this adverse event was unlikely to be causally related to anti-malarial therapy based on previous reports [3-7]. Younger age was associated with an increased risk of diarrhoea, but not vomiting, in both the AL and DP treatment arms. Repeat therapy within 17-28 days was associated with an increased risk of vomiting in the DP treatment arm (HR = 6.47, 95% CI 2.31-18.1, p < 0.001), but not the AL treatment arm. Among HIV-uninfected patients, there was no association between concomitant use of TS and the risk of diarrhoea or vomiting for both treatment arms. The independent effects of HIV in the absence of TS or ARV use could not be evaluated due to the small number of observations. Compared to HIV-uninfected not taking TS, concomitant use of TS and ARVs among HIV-infected patients was not associated with an increased risk of diarrhoea or vomiting in either treatment arm.

**Table 3:** Risk factors for adverse events due to diarrhoea or vomiting within 28 days following therapy

Risk category	Risk group	Diarrhoea				Vomiting			
		
		AL		DP		AL		DP	
		
		HR^† ^(95% CI)	p-value	HR^† ^(95% CI)	p-value	HR^† ^(95% CI)	p-value	HR^† ^(95% CI)	p-value
Age	>15-22 months	1.0 (reference)	-	1.0 (reference)	-	1.0 (reference)	-	1.0 (reference)	-
	>12-15 months	1.17 (0.56-2.46)	0.67	3.34 (1.39-8.02)	0.007	0.75 (0.10-5.58)	0.78	2.66 (0.80-8.85)	0.11
	>10-12 months	2.04 (1.09-3.85)	0.03	4.03 (1.90-8.53)	< 0.001	0.32 (0.04-2.86)	0.31	2.61 (0.66-10.3)	0.17
	4-10 months	2.11 (1.09-4.08)	0.03	3.22 (1.33-7.81)	0.01	1.14 (0.30-4.27)	0.84	1.08 (0.20-5.90)	0.93

Duration since last treatment	> 63 days or no prior treatment	1.0 (reference)	-	1.0 (reference)	-	1.0 (reference)	-	1.0 (reference)	-
	28-63 days	0.54 (0.34-0.86)	0.009	0.85 (0.47-1.52)	0.57	0.77 (0.24-2.42)	0.65	0.78 (0.30-2.02)	0.60
	17-28 days	0.68 (0.36-1.29)	0.24	1.33 (0.47-3.73)	0.59	0.71 (0.11-4.73)	0.72	6.63 (2.33-18.9)	< 0.001

HIV status,	HIV-uninfected not taking TS	1.0 (reference)	-	1.0 (reference)	-	1.0 (reference)	-	1.0 (reference)	-
TS use,	HIV-uninfected taking TS	0.78 (0.47-1.28)	0.32	1.26 (0.76-2.11)	0.37	1.04 (0.27-4.02)	0.95	1.30 (0.47-3.55)	0.62
ARV use*	HIV-infected taking TS and ARVs	0.33 (0.12-0.90)	0.03	0.46 (0.11-2.03)	0.31	1.77 (0.20-15.7)	0.61	1.85 (0.31-10.9)	0.50

^† ^HR = hazard ratio, generated using multivariate Cox proportional hazard models adjusted for repeated measures in the same patient

* HIV-infected patients not taking ARVs not included in the analysis due to small numbers (n = 16)

### Risk of adverse events due to anaemia and serious adverse events

Among 837 treatments with study drugs, 107 (13%) repeat haemoglobin measurements were made on the day of recurrent malaria and 8 (1%) had missing repeat haemoglobin measurements and were not included in the analysis of adverse events due to anaemia. There was no significant difference in the risks of adverse events due to anaemia in the AL and DP treatment arms (10% vs. 14%, p = 0.09).

Serious adverse events were rare. Out of 837 total treatments with study drugs, there were only 5 serious adverse events (two in the AL group and three in the DP group) and all were due to the development of severe anaemia, which was likely a consequence of malaria and not the study drugs. Of note, one patient developed severe anaemia twice following treatment with DP. This patient was removed from the study protocol because of initial concerns about a causal relationship between DP and severe anaemia. However, the patient developed a subsequent episode of malaria off-protocol that was treated with AL, and again developed severe anaemia, which resulted in his death. A blood sample taken during the anemic episode in the month prior to his death was direct Coombs test positive, indicative of immune-mediated haemolytic anaemia. Post-mortem this male child was found to be hemizygous for the G202A mutation, the predominant East African allele of glucose-6-phosphate dehydrogenase deficiency.

## Discussion

Results from this longitudinal randomized clinical trial suggest that both AL and DP are safe for treating uncomplicated malaria in young HIV-infected and uninfected infants and children. Adverse events were uncommon and generally of mild severity, with only cough, diarrhoea, vomiting, and anaemia occurring in more than 1% of treatments with study drugs. There were no significant differences in adverse events between the two treatment arms, although recent treatment with DP was associated with an increased risk of vomiting. Concomitant use of TS and ARVs were not associated with an increased risk of common adverse events.

This study contributes to a body of literature suggesting that AL and DP are both safe and well tolerated across a wide range of epidemiological settings. AL has been extensively studied in clinical trials primarily from Asia and Africa, and was added to the WHO Essential Medicines List in 2002. A review of AL safety and tolerability in 1,869 patients (33% children) from Asia reported gastro-intestinal complaints (nausea, vomiting, and diarrhoea), headache, and dizziness as the most commonly reported adverse events [16]. Several randomized clinical trials from Africa have reported adverse events associated with AL similar to those from Asia, with safety and tolerability profile found to be equivalent or superior to comparator regimens such as amodiaquine + sulphadoxine-pyrimethamine or amodiaquine + artesunate [17-22]. DP has also been shown to be very safe and well tolerated in studies from Asia and Africa. In a review of 2,600 patients from studies primarily done in Asia, gastrointestinal complaints were the most common adverse events associated with DP, with no evidence of severe drug toxicity [5]. In three clinical trials comparing AL (n = 597) versus DP (n = 613) in Africa using similar protocols, the risks of adverse events between these two treatment groups were similar [3,6,7]. In these studies a total of 13 serious adverse events (1.1%) were reported (AL = 4, DP = 9), and all were felt to be unrelated to the study drugs. The most severe adverse event in this trial, death due to presumed haemolytic anaemia, occurred in a child found to have G6PD deficiency. Haemolysis following treatment with DP in a child with G6PD deficiency has been described in a prior study from Laos [23]. However, it is unclear whether haemolytic anaemia is causally associated with DP or just a consequence of G6PD deficiency alone [23].

This study addresses some of the limitations from previously published studies on the safety and tolerability of AL and DP. First there is limited data on the safety of these drugs in children under 12 months of age, an important population in Africa. Study participants initiated ACT therapy at four months of age and over 50% of the treatments given for uncomplicated malaria were in children younger than 12 months. Both medications were well tolerated by infants, with diarrhoea the only adverse event associated with this younger age group, independent of the treatment given. Second, most anti-malarial clinical trials are limited to single episodes of uncomplicated malaria in the same patient, which precludes the ability to analyse the effect of repeated treatments. The longitudinal design used in this study allowed for the follow-up of children for up to one year, and observe the effects of repeated treatments of ACT drugs. Repeated therapy was found to be generally safe and well tolerated with the exception of a significantly higher risk of vomiting following repeat treatment with DP within 2-4 weeks of a previous dose. Although the extended half-life of piperaquine [24] provides a prolonged post-treatment prophylactic effect [3,6,7], repeat therapy with DP over a short period of time may increase the risk of adverse events and should be investigated further. This study also provides data on the safety and tolerability of ACT in unique patient populations including those taking TS prophylaxis and HIV-infected patients taking ARVs. Almost 40% of the patients were concomitantly taking TS when treated for malaria, however, this was not associated with an increased risk of adverse events. The concomitant use of ARVs and ACT is of greater concern because drug toxicities and the potential for drug interactions [25]. Artemether is metabolized via CYP3A4 to the more active compound dihydroartemisinin. Lumefantrine is also metabolized by CYP3A4. Less is known about the metabolism of piperaquine. There are three main classes of ARVs: protease inhibitors (PIs), non-nucleoside reverse transcriptase inhibitors (NNRTIs), and nucleoside reverse transcriptase inhibitors (NRTIs). PIs can be either potent inducers or inhibitors of CYP3A4. In a study of healthy subjects the PI lopinavir/ritonavir increased the lumefantrine area under the curve (AUC) by 193%, though no significant toxicities were reported [26]. NNRTIs can also be inducers or inhibitors of CYP3A4 with efavirenz primarily inhibiting CYP3A4 and nevirapine primarily inducing CYP3A4. Nucleoside reverse transcriptase inhibitors (NRTIs) are not thought to have clinical significant interactions with commonly used ACTs [25]. Prior studies have found an association between amodiaquine-based anti-malarial therapy and neutropaenia in HIV-infected children [27]. In this study, no association between the risk of adverse events and the concomitant use of ARVs was found. However, this study had a limited number of patients taking ARVs (n = 66), and only included NNRTI + NRTI containing regimens.

There were several limitations to this study. Firstly, although every effort was made to apply standardized definitions for adverse events, treatment assignment was not blinded and results were limited by largely subjective reports of symptoms from parents or guardians. Secondly, only haemoglobin levels were regularly measured during the period of malaria follow-up, preventing the detection other laboratory associated adverse events. Finally, this study was not powered to specifically test for hypothesis of differences in the risk of adverse events between the various subgroups. Therefore, the possibility of type II errors cannot be ruled out, especially in those subgroups with small samples sizes such as HIV-infected children taking ARVs.

## Conclusion

In summary, both AL and DP were safe and well tolerated for the treatment of uncomplicated malaria in a cohort of children uniquely characterized by their young age, repeated therapy, and the inclusion of HIV-exposed and HIV-infected patients. However, the occurrence of haemolytic anaemia leading to death in a G6PD deficient patient highlights the importance of monitoring for rare, but serious adverse events. As the use of ACT is scaled up in Africa, continued evaluation of the safety and tolerability of these drugs in diverse patient populations is essential.

## Competing interests

The authors declare that they have no competing interests.

## Authors' contributions

GD and EA supervised the clinical studies. SK and GD analysed the data. All authors contributed to the drafting of the manuscript.
